# Relation of Age, Gender, and Bone Mass to Circulating Sclerostin Levels in Women and Men

**DOI:** 10.1002/jbmr.217

**Published:** 2010-08-18

**Authors:** Ulrike I Mödder, Kelley A Hoey, Shreyasee Amin, Louise K McCready, Sara J Achenbach, B Lawrence Riggs, L Joseph Melton, Sundeep Khosla

**Affiliations:** Endocrine Research Unit, College of Medicine, Mayo Clinic Rochester, MN, USA

**Keywords:** SCLEROSTIN, OSTEOPOROSIS, AGING

## Abstract

Sclerostin is a potent inhibitor of Wnt signaling and bone formation. However, there is currently no information on the relation of circulating sclerostin levels to age, gender, or bone mass in humans. Thus we measured serum sclerostin levels in a population-based sample of 362 women [123 premenopausal, 152 postmenopausal not on estrogen treatment (ET), and 87 postmenopausal on ET] and 318 men, aged 21 to 97 years. Sclerostin levels (mean ± SEM) were significantly higher in men than women (33.3 ± 1.0 pmol/L versus 23.7 ± 0.6 pmol/L, *p* < .001). In pre- and postmenopausal women not on ET combined (*n* = 275) as well as in men, sclerostin levels were positively associated with age (*r* = 0.52 and *r* = 0.64, respectively, *p* < .001 for both). Over life, serum sclerostin levels increased by 2.4- and 4.6-fold in the women and men, respectively. Moreover, for a given total-body bone mineral content, elderly subjects (age ≥ 60 years) had higher serum sclerostin levels than younger subjects (ages 20 to 39 years). Our data thus demonstrate that (1) men have higher serum sclerostin levels than women, (2) serum sclerostin levels increase markedly with age, and (3) compared with younger subjects, elderly individuals have higher serum sclerostin levels for a given amount of bone mass. Further studies are needed to define the cause of the age-related increase in serum sclerostin levels in humans as well as the potential role of this increase in mediating the known age-related impairment in bone formation. © 2011 American Society for Bone and Mineral Research.

## Introduction

Considerable work over the past decade has established the Wnt/β-catenin signaling pathway as a major regulator of bone mass.(1,2) Activation of this pathway results in an expansion of osteoprogenitor cells as well as reduced apoptosis of mature osteoblasts, leading to increased bone formation and potentially marked increases in bone mass.(1,2) The effects of Wnt ligands on the canonical signaling pathway involving β-catenin signaling are mediated by binding of these ligands to a seven-transmembrane domain-spanning frizzled receptor and either of two coreceptors, low-density lipoprotein receptor–related proteins (LRP) 5 or 6.(1,2)

Sclerostin is a secreted Wnt antagonist produced almost exclusively by osteocytes that regulates bone mass by binding to LRP5 and LRP6 to inhibit the canonical Wnt/β-catenin signaling pathway.(1,3–6) The biologic importance of sclerostin in humans is highlighted by sclerosteosis and van Buchem disease, two genetic diseases associated with markedly increased bone mass.(7–10) Sclerosteosis is caused by a mutation in the gene encoding sclerostin, *SOST*, that leads to improper splicing of the *SOST* mRNA,(7,8) whereas van Buchem disease is due to a deletion of an enhancer element downstream of the *SOST* gene.(9,10) In addition to these human diseases, mice with knockout of the *Sost* gene also have increased bone-formation rates at trabecular, endocortical, and periosteal surfaces, as well as increased bone mass.(11) Thus, given the compelling evidence for sclerostin as a major regulator of Wnt signaling and bone mass, an antisclerostin neutralizing antibody has been developed recently as a novel anabolic treatment for osteoporosis.(12)

Despite growing understanding of the basic biology of Wnt signaling and its regulation by sclerostin, there are currently sparse data on assessment of circulating sclerostin levels in humans. Using an in-house Amgen immunoassay (Thousand Oaks, CA, USA), Mirza and colleagues(13) demonstrated that sclerostin could be measured in peripheral serum and was higher in 20 postmenopausal than in an equal number of premenopausal women. These investigators also found that in the postmenopausal women there were significant negative correlations between sclerostin levels and the free estrogen (E) index and parathyroid hormone (PTH) levels. In recent studies, we used a commercially available immunoassay (Biomedica, Vienna, Austria) to demonstrate that E treatment decreased circulating sclerostin levels in women and that selective elimination of E, but not testosterone (T), production in men resulted in increased serum sclerostin levels.(14) In addition, Gaudio and colleagues(15) have used the same immunoassay to demonstrate increased circulating sclerostin levels in long-term immobilized patients. Thus, given the availability of a validated immunoassay for sclerostin, we sought in this study to measure serum sclerostin levels in a population-based sample of women and men in order to define possible relations of sclerostin levels to age and gender, as well as bone mass, microstructure, sex steroid levels, and bone turnover markers.

## Methods

### Study subjects

We recruited subjects from an age-stratified random sample of Rochester, MN, residents who were selected using the medical records linkage of the Rochester Epidemiology Project.(16) This population is highly characteristic of the U.S. white population, but blacks and Asians are underrepresented.(17) The sample spanned ages from 21 to 97 years and included 362 women and 318 men. Reflecting the ethnic composition of the community, 98% of the subjects were white. There were 123 premenopausal women and 239 postmenopausal women; of the postmenopausal women, 87 were on some form of estrogen therapy (ET, defined as oral or transdermal E preparations with or without a progestin). All studies were approved by the Mayo Institutional Review Board, and written informed consent was obtained from all subjects prior to evaluation.

### Study protocol

Subjects were evaluated at the outpatient Clinical Research Unit following an overnight fast. They consumed their habitual diet the day prior to study without any dietary restrictions. Following a blood draw, the subjects underwent the various imaging procedures described below.

### Total-body dual-energy X-ray absorptiometry (DXA)

This study was designed originally to focus on volumetric quantitative computed tomographic (QCT) parameters and did not include site-specific DXA measures of the spine or hip. However, the subjects did have a total-body DXA performed (Prodigy, GE Medical Systems, Madison, WI, USA) using software Version 6.10.029. From this, we derived the total-body bone mineral content (TBBMC) and total-body bone mineral density (TBBMD). In addition, we were able to obtain the spine region areal BMD (aBMD) from the total-body scans. We have shown previously that such scans are equivalent to dedicated lumbar spine DXA measurements in women, with *r* = 0.92 and an error in predicting lumbar spine BMD of 6.5%.(18)

### Central QCT

As described previously,(16,19) single-energy CT scans were made at the lumbar spine and proximal femur with a multidetector Light Speed QX-I Scanner (GE Medical Systems, Wakesha, WI, USA). Calibration standards scanned with the patient were used to convert CT numbers directly to equivalent volumetric BMD (vBMD) in mg/cm^3^.(20) To study age- and sex-specific structural changes in bone mineral distribution and structure, we developed software for the analysis of bone structure, geometry, and volumetric density from the CT images, specific details of which have been described previously.(19) To validate our image-processing algorithm, we made 10 scans of the European spine phantom, which is composed of hydroxyapatite.(21) The correlation between bone density results determined by our algorithm and that of the spine phantom was *r* = 0.998; using scans of L_2_ from the phantom over 10 days, vBMD was estimated to have a coefficient of variation (CV) of 0.7%.

### HRpQCT

Details regarding the high-resolution peripheral quantitative computed tomographic (HRpQCT) imaging used in this cohort have been reported previously(22) and are summarized briefly here. Owing to the lack of availability of this new instrument initially, the HRpQCT measurements were done about 2 years after the other measurements. The HRpQCT scans were obtained in 237 (86%) of the 275 women not on ET used in this study (107 premenopausal women and 130 postmenopausal women) and in 282 (89%) of the 318 men. The nondominant wrist (or in the case of a prior wrist fracture, the nonfractured wrist) was scanned using a prototype of the XtremeCT (Scanco Medical AG, Bassersdorf, Switzerland). The in vivo measurement protocol included the acquisition of a 3D stack of 116 high-resolution QCT slices at the distal end of the radius using an effective energy of 40 keV, slice thickness of 89 µm, field of view of 90 mm, image matrix of 1024 × 1024 pixels, and pixel size of 89 µm.

The processing and analysis of the images also have been described extensively and validated.(23–26) Briefly, bone volume/total volume (BV/TV) is first derived from the trabecular vBMD. Recognizing that individual trabeculae will not be resolved at their correct thickness owing to partial-volume effects, a thickness-independent structure extraction is employed to assess trabecular microarchitecture. To this end, the 3D ridges (the center points of the trabeculae) are detected in the gray-level images,(24) and trabecular number (Tb.N, 1/mm) then is taken as the inverse of the mean spacing of the ridges.(25) Trabecular thickness (Tb.Th, mm) then is derived as BV/TV ÷ Tb.N, and trabecular separation (Tb.Sp, mm) is derived as (1 – BV/TV) ÷ Tb.N, as is done in standard histomorphometry.(27) The validity of this approach has been tested rigorously by comparing the HRpQCT methodology with 28-µm resolution micro–computed tomography (µCT),(26) with very high correlation (correlation coefficients of 0.96–0.99) between the µCT and HRpQCT measurements. The key point is that HRpQCT resolution has to be sufficient to adequately resolve the distance between the trabecular ridges (1/Tb.N, or about 300 to 500 µm), not necessarily to resolve individual trabeculae (∼100 µm or less).

### Serum measurements

Serum sclerostin levels were measured using a recently available quantitative sandwich enzyme-linked immunosorbent assay (ELISA) developed by Biomedica and obtained from ALPCO (Salem, NH, USA).(14,15,28) This assay uses a polyclonal goat anti-human sclerostin antibody as a capture antibody and a biotin-labeled mouse monoclonal antisclerostin antibody for detection. The interassay CV was 4%, and the lower limit of detection was 3.6 pmol/L. Results of further validation of this assay are shown in Table 1. Thus, spiking of control serum with two known amounts of a sclerostin standard resulted in values that were 96% and 98%, respectively, of the predicted values. Conversely, serial dilutions (1:2 and 1:4) of control serum resulted in values that were 105% and 103%, respectively, of the predicted values (Table 1). Results for the sclerostin measurements are reported throughout in picomoles per liter (multiply by 22.7 to convert to picograms per milliliter).

**Table 1:** Characterization of the Sclerostin Assay by Spiking and Serial Dilutions of Control Serum

	Measured sclerostin, pmol/L	Predicted sclerostin, pmol/L	Measured as percent of predicted
Spike			
Control serum A	14.8	—	—
Control serum A + 2 µL 80 pmol/L standard	20.4	21.3	96%
Control serum A + 5 µL 80 pmol/L standard	30.3	31.2	97%
Serial dilution			
Control serum B	16.1	—	—
1:2 Dilution	8.5	8.1	105%
1:4 Dilution	4.1	4.0	103%

Serum PTH was measured using a two-site immunoassay for intact PTH (Diagnostic Products Corporation, Los Angeles, CA, USA; interassay CV < 13%). Serum osteocalcin (OC) was measured using a two-site immunoradiometric assay (CIS-US, Bedford, MA, USA; interassay CV = 8%). Serum N-terminal propeptide of type I collagen (P1NP) was measured by radioimmunoassay (DiaSorin, Stillwater, MN, USA; interassay CV < 9%). Serum bone alkaline phosphatase (B-ALP) was measured using an immunoassay from Quidel Corporation (San Diego, CA, USA; interassay CV < 8%). Serum cross-linked C-telopeptide of type I collagen (CTX) was measured using an ELISA (Nordic Biosciences, Herlev, Denmark; interassay CV < 10%), and serum tartrate-resistant acid phosphatase isoform type 5b (TRACP 5b) also was measured by ELISA (Immunodiagnostic Systems, Scotsdale, AZ, USA; interassay CV < 14%). Serum E_2_ and T were measured using LC-MS/MS (API 5000, Applied Biosystems-MDS Sciex, Foster City, CA, USA), as described previously.(29) Values as low as 1.25 pg/mL for E_2_ and 1 ng/dL for T were detectable by this method, with interassay CVs of 8% and 6% for E_2_ and T, respectively. The non–sex hormone–binding globulin–bound, biologically active (bio) fraction of E_2_ and T was measured as described previously(30); interassay CVs for each were less than 12%.

### Statistical analysis

Sclerostin levels were summarized as means ± SEMs. Two-sample *t* tests were used for comparisons between groups. Unadjusted and age-adjusted Pearson correlation coefficients were used to assess relationships between serum sclerostin levels with the various bone density variables, microstructural parameters, and biochemical markers. Changes in sclerostin levels between the ages of 20 and 90 years were based on predicted values from a linear model. Multivariable linear regression models were developed to calculate the least-squares (LS) means of sclerostin after adjusting for TBBMC. A *p* value of less than .05 was considered significant. All analyses were performed using SAS Version 9 (SAS Institute, Cary, NC, USA) and Splus (TIBCO Corporation, Palo Alto, CA, USA).

## Results

**Fig. 1:**
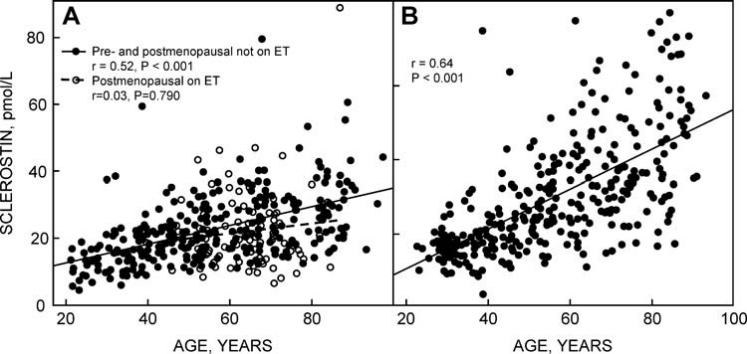
Serum sclerostin levels versus age in (*A*) women and (*B*) men. Correlation coefficients are as noted.

Figure 1*A* shows serum sclerostin levels as a function of age in pre- and postmenopausal women not on ET combined as well as postmenopausal women on ET, and Fig. 1*B* shows the analogous plot for all men. Sclerostin levels were significantly associated with age in the pre- and postmenopausal women not on ET combined (*r* = 0.52, *p* < .001) and in the men (*r* = 0.64, *p* < .001). Over life, serum sclerostin levels increased by 2.4- and 4.6-fold in the women and men, respectively. Since the route of metabolism and/or clearance of circulating sclerostin (eg, hepatic versus renal) is currently unknown, we also adjusted for possible effects of age-related reductions in creatinine clearance. In this analysis, the creatinine clearance–adjusted correlations with age were 0.47 and 0.56 in women and men, respectively (*p* < .001 for both), and the adjusted increases with age were 2.4- and 4.1-fold in women and men, respectively.

Comparing the pre- and postmenopausal women not on ET with the men, we found that serum sclerostin levels were significantly higher in men than in women (33.3 ± 1.0 pmol/L versus 23.7 ± 0.6 pmol/L, *p* < .001). Among the postmenopausal women, serum sclerostin levels were significantly lower in the women on ET than in the women not on ET (22.7 ± 1.2 pmol/L versus 27.8 ± 0.8 pmol/L, *p* < .001; Fig. 1*A*).

**Fig. 2:**
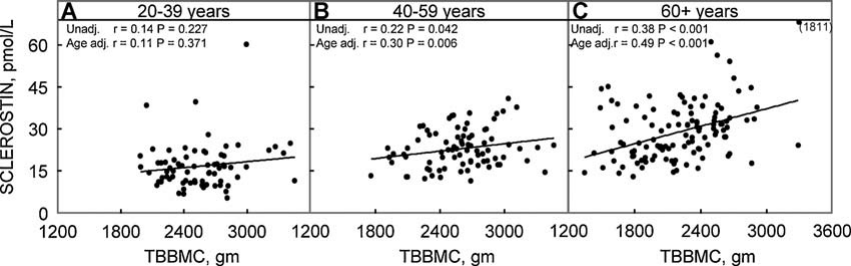
Serum sclerostin levels versus total-body bone mineral content (TBBMC) in young (ages 20 to 39 years), middle-aged (40 to 59 years), and elderly (60+ years) women. Unadjusted and age-adjusted correlation coefficients are as noted.

**Fig. 3:**
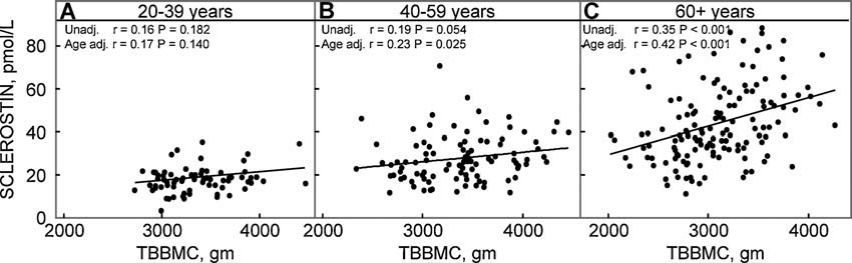
Serum sclerostin levels versus total-body bone mineral content (TBBMC) in young (ages 20 to 39 years), middle-aged (40 to 59 years), and elderly (60+ years) men. Unadjusted and age-adjusted correlation coefficients are as noted.

Since sclerostin is produced almost exclusively by osteocytes,(1,3,5,6) we next examined the relation between serum sclerostin levels and total-body bone mass, estimated using TBBMC by DXA. In order to reduce confounding by age, we separated the subjects into three groups: young (ages 20 to 39 years), middle-aged (40 to 59 years), and elderly (60+ years). Because of the effects of ET on sclerostin levels noted earlier, we used only women not on ET in this analysis. As shown in Fig. 2*A*, there was no association between TBBMC and serum sclerostin levels in young women, a modest association was present in middle-aged women (Fig. 2*B*), and the strongest positive correlations were noted in elderly women (Fig. 2*C*). As also evident, for a given level of TBBMC, sclerostin levels were higher in elderly compared with young women. To formally evaluate this, we compared serum sclerostin levels adjusted for TBBMC. These were significantly higher (*p* < .001) in the elderly women (29.9 ± 0.9 pmol/L) than in the young women (15.1 ± 1.2 pmol/L). Figure 3*A–C* shows the analogous plots for serum sclerostin levels versus TBBMC in the young, middle-aged, and elderly men. As in the case of the women, there was no association between TBBMC and serum sclerostin levels in young men (Fig. 3*A*), a modest association was present in middle-aged men (Fig. 3*B*), and the strongest positive correlations were noted in elderly men (Fig. 3*C*). As for the women, TBBMC-adjusted sclerostin levels were significantly higher (*p* < .001) in the elderly men (45.1 ± 1.2 pmol/L) than in the young men (17.5 ± 1.7 pmol/L).

We next assessed the relation between serum sclerostin levels and bone density and microstructural parameters in the three groups of women and men, as defined earlier (Table 2). None of these parameters was correlated with serum sclerostin levels in the young women. In the middle-aged women, unadjusted and age-adjusted TBBMD and spine aBMD were positively associated with serum sclerostin levels. By contrast, the bone density and microstructural parameters generally were positively associated with serum sclerostin levels in the elderly women (except Tb.Sp, which showed an inverse association). The overall pattern was very similar in the men, with elderly men showing positive associations of all the bone density and microstructural parameters with sclerostin levels (again, with the exception of Tb.Sp, which showed an inverse association).

**Table 2:** Correlation Coefficients (Unadjusted/Age-Adjusted) Between Bone Density and Microstructural Parameters and Serum Sclerostin Levels

	Women			Men		
		
Age group (years)	20–39	40–59	60+	20–39	40–59	60+
DXA						
TBBMD	0.04/0.04	0.29**/0.34**	0.38***/0.49***	0.29*/0.31**	0.13/0.14	0.42***/0.50***
Spine BMD	0.14/0.11	0.23*/0.28*	0.42***/0.46***	0.22/0.19	0.23*/0.22*	0.46***/0.44***
QCT						
FN total vBMD	0.02/0.11	0.08/0.20	0.20*/0.33***	−0.00/0.05	0.13/0.19	0.24**/0.36***
FN cortical vBMD	0.09/0.13	0.06/0.17	0.19*/0.23*	−0.01/0.02	0.14/0.20	0.16/0.19*
FN trabecular vBMD	−0.08/0.04	0.09/0.19	0.16/0.34***	−0.06/0.00	0.07/0.14	0.26**/0.42***
Vertebral trabecular vBMD	−0.12/−0.09	0.04/0.18	0.24**/0.38***	0.11/0.12	0.02/0.08	0.28***/0.44***
Radius HRpQCT						
Cortical vBMD	−0.00/−0.01	−0.11/−0.07	0.07/0.14	0.11/0.12	0.17/0.18	0.08/0.25**
BV/TV	−0.07/−0.08	0.03/0.08	0.25*/0.29**	−0.05/−0.05	0.08/0.12	0.35***/0.37***
Tb.N	0.01/0.02	−0.01/−0.00	0.31**/0.39***	0.34**/0.33**	0.13/0.13	0.31***/0.39***
Tb.Th	−0.07/−0.09	−0.00/0.06	0.18/0.19	−0.24/−0.24	0.00/0.04	0.26**/0.26**
Tb.Sp	−0.01/−0.02	0.02/−0.01	−0.29**/−0.36***	−0.24*/−0.24	−0.14/−0.16	−0.35***/−0.42***

TBBMD = total-body bone mineral density; FN = femoral neck; vBMD = volumetric BMD; BV/TV = bone volume/tissue volume; Tb.N = trabecular number; Tb.Th = trabecular thickness; Tb.Sp = trabecular separation.

* *p* < .05.

** *p* < .01.

*** *p* < .001.

Table 3 shows the unadjusted and age-adjusted correlations among biochemical markers of bone turnover, hormone levels, and sclerostin levels in the three groups. Of interest, serum bone-formation markers (B-ALP and P1NP but not OC) were inversely associated with sclerostin levels in the elderly women; no associations were present with the bone-formation markers in the elderly men. Serum CTX also was inversely associated with sclerostin levels in the elderly women but not men. Serum E_2_ or T levels were not associated with sclerostin levels in any group other than elderly men, in whom serum E_2_ and bio E_2_ levels were positively correlated with sclerostin levels.

**Table 3:** Correlation Coefficients (Unadjusted/Age-Adjusted) Between Serum Sclerostin Levels and Biochemical Markers of Bone Turnover and Serum Hormone Levels

	Women			Men		
		
Age group (years)	20–39	40–59	60+	20–39	40–59	60+
OC	−0.05/0.07	0.13/0.10	−0.15/−0.18	−0.08/−0.06	0.04/0.12	0.02/−0.06
B-ALP	−0.24*/−0.16	−0.07/−0.12	−0.26**/−0.29**	0.04/0.07	0.02/0.07	−0.07/−0.13
P1NP	−0.07/0.03	0.08/0.04	−0.27**/−0.27**	0.03/0.09	−0.04/0.03	−0.07/−0.05
CTX	−0.11/−0.01	0.11/0.06	−0.19*/−0.22*	−0.24*/−0.20	−0.11/−0.06	−0.02/−0.05
TRAP 5b	−0.24*/−0.22	0.13/0.07	−0.06/−0.12	−0.23*/−0.20	0.22*/0.20	−0.20*/−0.23**
PTH	0.07/0.00	−0.06/−0.08	−0.11/−0.13	0.14/0.14	0.14/0.07	0.20*/0.13
E_2_	−0.05/−0.20	−0.02/0.07	0.02/0.02	0.08/0.14	0.03/−0.04	0.27**/0.27**
Bio E_2_	−0.03/−0.07	−0.00/0.10	0.09/0.12	0.02/0.09	0.01/−0.01	0.20*/0.30***
T	−0.05/−0.02	−0.01/0.06	0.20*/0.16	−0.11/−0.12	−0.15/−0.14	−0.03/−0.01
Bio T	0.18/0.16	0.15/0.18	0.17/0.15	−0.09/−0.05	−0.21*/−0.16	−0.08/0.09

OC = osteocalcin; B-ALP = bone alkaline phosphatase; P1NP = N-terminal propeptide of type I collagen; CTX = cross-linked C-telopeptide of type I collagen; TRAP 5b = tartrate-resistant acid phosphatase isoform type 5b; PTH = parathyroid hormone; E_2_ = estradiol; bio = bioavailable; T = testosterone.

* *p* < .05.

** *p* < .01.

*** *p* < .001.

## Discussion

In this study, we measured serum sclerostin levels in a population-based sample of women and men using a commercial immunoassay for sclerostin that has been applied in previous studies by us(14) and by others.(15,28) In addition, we further validated this assay in our laboratory using spiking and dilution experiments. Our data demonstrate that serum sclerostin levels increase markedly with age in women and even more so in men. Given that sclerostin is produced almost exclusively by osteocytes,(1,3,5,6) this observation would be consistent with increased skeletal sclerostin production with aging in humans (see below). While we cannot exclude the possibility that the age-related increase in circulating sclerostin levels is due, at least in part, to reduced clearance of the protein, the increase in sclerostin levels with age was minimally altered following adjustment for effects of age-related reductions in creatinine clearance.

Because of the strong correlation with age, we sought to minimize the potential confounding effects of age by dividing our cohort into young, middle-aged, and elderly subjects. Using TBBMC as a surrogate for total skeletal mass, we found that while TBBMC was not associated with serum sclerostin levels in young women or men, TBBMC was positively associated with sclerostin levels in the elderly subjects of both sexes. Since sclerostin is a potent inhibitor of bone formation,(1,3) one might have predicted the opposite, namely, that serum sclerostin levels would be inversely associated with TBMMC. The fact that we observed a similar pattern of lack of association in young subjects and positive associations in the elderly subjects between serum sclerostin levels and virtually all the other skeletal parameters assessed (spine aBMD, QCT vBMD, bone microstructural variables by HRpQCT) argues that the correlations we noted were not due to chance and may reflect changes occurring with aging in skeletal sclerostin production. Specifically, our data also demonstrate that for any given TBBMC, and presumably an equivalent number of osteocytes, serum sclerostin levels are higher in the elderly compared with the young subjects. This suggests that with aging, there is increased sclerostin production by individual osteocytes. While our data are consistent with this hypothesis, further animal and human studies addressing this important issue are needed. Moreover, whether increased skeletal sclerostin production with aging explains, at least in part, the known age-related impairment in bone formation(31) warrants further investigation.

We also observed that at any age, serum sclerostin levels were higher in men than in women. The reasons for this are unclear, but to the extent that circulating sclerostin levels might reflect total-body skeletal mass, the larger skeleton in men simply may produce and release more sclerostin into the circulation. We also found that among postmenopausal women, serum sclerostin levels were significantly lower in women on ET compared with women not on ET. This finding is consistent with recent data from our group(14) showing that E treatment of postmenopausal women for 4 weeks leads to a 27% decrease in serum sclerostin levels. Unlike the findings of Mirza and colleagues,(13) however, we did not observe any correlations between serum sclerostin and estradiol (total or bioavailable) levels in any of the three groups of women. In fact, in the men, we found the opposite, in that serum estradiol levels were positively associated with sclerostin levels. In our recent study involving 59 elderly men, increases in serum sclerostin levels following combined T and E deficiency were prevented by E treatment alone but not T treatment without E.(14) Thus, while our previous interventional data clearly demonstrate that E reduces serum sclerostin levels,(14) the correlation analyses in this study are not consistent with this observation, suggesting that there may be additional confounders in the relationship between E and sclerostin levels that we may not have accounted for in this analysis.

Given the previously noted effects of sclerostin in inhibiting bone formation,(1,3) bone-formation markers would be expected to be inversely associated with sclerostin levels. This was indeed the case in the elderly women but not in the men. The reasons for this gender difference are unclear at present. Since the specific dose relationship between sclerostin and inhibition of bone formation is not known, it is possible, for example, that the higher sclerostin levels in the elderly men (to the extent that these levels reflect skeletal sclerostin production) might be beyond some biologic saturation point for sclerostin effects on bone formation, leading to the lack of any correlation between these levels and bone-formation markers in the men. Additional studies assessing the dose relationship between sclerostin levels (and sclerostin production) and bone formation are needed to test this hypothesis.

In summary, our study represents the first population-based assessment of circulating sclerostin levels in women and men over a broad age range using a well-validated immunoassay. These findings point to the need for further studies examining the mechanisms for the age-related increase in serum sclerostin levels we observed in both sexes and the potential role of increased skeletal sclerostin production in mediating the known age-related decrease in bone formation in humans.(31)

## References

[1] Barons R, Rawadi G (2007). Targeting the Wnt/β-catenin pathway to regulate bone formation in the adult skeleton. Endocrinology..

[2] Krishnan V, Bryant HU, MacDougald OA (2006). Regulation of bone mass by Wnt signaling. J Clin Invest..

[3] van Bezooijen RL, Roelen BAJ, Visser A (2004). Sclerostin is an osteocyte-expressed negative regulator of bone formation, but not a classical BMP antagonist. J Exp Med..

[4] Semenov M, Tamai K, He X (2005). *SOST* is a ligand for LRP5/LRP6 and a Wnt signaling inhibitor. J Biol Chem..

[5] Poole KES, van Bezooijen RL, Loveridge N (2005). Sclerostin is a delayed secreted product of osteocytes that inhibits bone formation. FASEB J..

[6] van Bezooijen RL, ten Dijke P, Papapoulos SE, Lowik CW (2005). SOST/sclerostin, an osteocyte-derived negative regulator of bone formation. Cytokine Growth Factor Rev..

[7] Brunkow ME, Gardner JC, Van Ness J (2001). Bone dysplasia sclerosteosis results from loss of the SOST gene product, a novel cystine knot-containing protein. Am J Hum Genet..

[8] Balemans W, Ebeling M, Patel N (2001). Increase bone density in sclerosteosis is due to the deficiency of a novel secreted protein (SOST). Hum Mol Genet..

[9] Staehling-Hampton K, Proll S, Paeper BW (2002). A 52-kb deletion in the SOST-MEOX1 intergenic region on 17q12-q21 is associated with van Buchem disease in the Dutch population. Am J Med Genet..

[10] Balemans W, Patel N, Ebeling M (2002). Identification of a 52kb deletion downstream of the SOST gene in patients with van Buchem disease. J Med Genet..

[11] Li X, Ominsky MS, Niu Q-T (2008). Targeted deletion of the sclerostin gene in mice results in increased bone formation and bone strength. J Bone Miner Res..

[12] Li X, Ominsky MS, Warmington KS (2009). Sclerostin antibody treatment increases bone formation, bone mass, and bone strength in a rat model of postmenopausal osteoporosis. J Bone Miner Res..

[13] Mirza FS, Padhi ID, Raisz LG, Lorenzo JA (2010). Serum sclerostin levels negatively correlate with parathyroid hormone levels and free estrogen index in postmenopausal women. J Clin Endocrinol Metab..

[14] Modder UIL, Clowes JA, Hoey K (2011). Regulation of circulating sclerostin levels by sex steroids in women and men. J Bone Miner Res..

[15] Gaudio A, Pennisi P, Bratengeier C (2010). Increased sclerostin serum levels associated with bone formation and resorption markers in patients with immobilization-induced bone loss. J Clin Endocrinol Metab..

[16] Riggs BL, Melton LJ, Robb RA (2004). Population-based study of age and sex differences in bone volumetric density, size, geometry, and structure at different skeletal sites. J Bone Miner Res..

[17] Melton LJ (1996). History of the Rochester Epidemiology Project. Mayo Clin Proc..

[18] Melton LJ, Looker AC, Shepherd JA (2005). Osteoporosis assessment by whole body region vs. site-specific DXA. Osteoporos Int..

[19] Camp JJ, Karwoski RA, Stacy MC (2004). A system for the analysis of whole-bone strength from helical CT images. Proceedings of SPIE..

[20] Cann CE (1988). Quantitative CT for determination of bone mineral density: a review. Radiology..

[21] Kalender WA, Felsenberg D, Genant HK, Fischer M, Dequeker J, Reeve J (1995). The European spine phantom: a tool for standardization and quality control in spinal bone mineral measurements by DXA and QCT. Eur J Radiol..

[22] Khosla S, Riggs BL, Atkinson EJ (2006). Effects of sex and age on bone microstructure at the ultradistal radius: a population-based noninvasive in vivo assessment. J Bone Miner Res..

[23] Muller R, Hahn M, Vogel M, Delling G, Ruegsegger P (1996). Morphometric analysis of noninvasively assessed bone biopsies: comparison of high-resolution computed tomography and histologic sections. Bone..

[24] Laib A, Hilderbrand T, Hauselmann HJ, Ruegsegger P (1997). Ridge number density: a parameter for in vivo bone structure analysis. Bone..

[25] Laib A, Hauselmann HJ, Ruegsegger P (1998). In vivo high resolution 3D-QCT of the human forearm. Technol Health Care..

[26] Laib A, Ruegsegger P (1999). Calibration of trabecular bone structure measurements of in vivo three-dimensional peripheral quantitative computed tomography with 28-microm-resolution microcomputed tomography. Bone..

[27] Parfitt AM, Mathews CHE, Villaneuva AR, Kleerekoper M, Frame B, Rao DS (1983). Relationships between surface, volume, and thickness of iliac trabecular bone in aging and in osteoporosis. J Clin Invest..

[28] Terpos E, Christoulas D, Katodritou E (2009). High serum sclerostin correlates with advanced stage, increased bone resorption, reduced osteoblast function, and poor survival in newly-diagnosed patients with multiple myeloma. Blood..

[29] Khosla S, Amin S, Singh RJ, Atkinson EJ, Melton LJ, Riggs BL (2008). Comparison of sex steroid measurements in men by immunoassay versus mass spectroscopy and relationships with cortical and trabecular volumetric bone mineral density. Osteoporos Int..

[30] Khosla S, Melton LJ, Atkinson EJ, O'Fallon WM, Klee GG, Riggs BL (1998). Relationship of serum sex steroid levels and bone turnover markers with bone mineral density in men and women: A key role for bioavailable estrogen. J Clin Endocrinol Metab..

[31] Lips P, Courpron P, Meunier PJ (1978). Mean wall thickness of trabecular bone packets in the human iliac crest: changes with age. Calcif Tissue Res..

